# An Ultrasensitive Electrochemical Immunosensor for Alpha-Fetoprotein Using an Envision Complex-Antibody Copolymer as a Sensitive Label

**DOI:** 10.3390/ma5122757

**Published:** 2012-12-11

**Authors:** Ping Xiong, Ning Gan, Yuting Cao, Futao Hu, Tianhua Li, Lei Zheng

**Affiliations:** 1 The State Key Laboratory Base of Novel Functional Materials and Preparation Science, Faculty of Material Science and Chemical Engineering, Ningbo University, Ningbo 315211, China; E-Mails: 519791696@qq.com (P.X.); caoyuting@nbu.edu.cn (Y.C.); hufutao@nbu.edu.cn (F.H.); litianhua@nbu.edu.cn (T.L.); 2 Department of Laboratory Medicine, Nanfang Hospital, Southern Medical University, Guangzhou 510515, China

**Keywords:** envision antibody complex, alfa-fetoprotein, signal amplification, electrochemical immunosensor

## Abstract

A novel strategy is presented for sensitive detection of alfa-fetoprotein (AFP), using a horseradish peroxidase (HRP)-functionalized Envision antibody complex (EVC) as the label. The Envision-AFP signal antibody copolymer (EVC-AFP Ab2) was composed of a dextran amine skeleton anchoring more than 100 molecules of HRP and 15 molecules of secondary antibody, and acted as a signal tag in the immunosensor. The sensor was constructed using the following steps: First, gold electrode (GE) was modified with nano-gold (AuNPs) by electro-deposition in HAuCl_4_ solution. The high affinity of the AuNPs surface facilitates direct formation of a self-assembled thiolated protein G layer. Next, the coated GE was incubated in a solution of AFP capture antibody (AFP Ab1); these antibodies attach to the thiolated protein G layer through their non-antigenic regions, leaving the antigen binding sites for binding of target analyte. Following a sandwich immunoreaction, an EVC-AFP Ab2-AFP-AFP Ab1 immunocomplex was formed on the electrode surface, allowing large amounts of HRP on the complex to produce an amplified electrocatalytic current of hydroquinone (HQ) in the presence of hydrogen peroxide (H_2_O_2_). Highly amplified detection was achieved, with a detection limit of 2 pg/mL and a linear range of 0.005–0.2 ng/mL for AFP in 10 μL undiluted serum; this is near or below the normal levels of most cancer biomarker proteins in human serum. Measurements of AFP in the serum of cancer patients correlated strongly with standard enzyme-linked immunosorbent assays. These easily fabricated EVC-modified immunosensors show excellent promise for future fabrication of bioelectronic arrays. By varying the target biomolecules, this technique may be easily extended for use with other immunoassays, and thus represents a versatile design route.

## 1. Introduction

Alfa-fetoprotein (AFP), a glycoprotein with a molecular weight of about 70,000 Da, is used as a serum-based marker for several types of malignant neoplasm, including hepatic and yolk sac tumors [1,2,3]. Sensitive detection of AFP is crucial for many areas of modern biochemical and biomedical research, providing opportunities for understanding the fundamental biological processes involved in disease progression, and for monitoring the patient response to therapy. Conventional immunoassay methods, including the enzyme-linked immunosorbent assay (ELISA) [4,5], radioimmunoassay [6,7], fluorescence immunoassay [8,9], chemiluminescence assay [10,11], electrophoretic immunoassay [12], mass spectrometric immunoassay [13,14] and immune-polymerase chain reaction assay [15], allow for reliable predictions. ELISA is widely used as an immunoassay method in clinical laboratories. However, the concentrations of tumor-related proteins are very low in early-stage cancer. The serum AFP level in healthy humans is less than 20 ng/mL [16], below the ELISA detection limit. Hence, new methods are required that can promptly and efficiently monitor tumor-related proteins. Electrochemical immunosensors, which are based on the specificity of an antigen-antibody interaction, have attracted extensive interest because of their intrinsic advantages, such as low cost, high sensitivity, simple instrumentation and excellent compatibility with miniaturization technologies [17]. The electrochemical method is also more suitable for analyzing turbid samples. Potential, capacitive and amperometric electrochemical immunosensors have been widely used for direct and indirect immune-analysis. Among these, amperometric immunosensors have attracted considerable interest because they have favorable features that include a fast response time, a wide linear range and a low limit of detection [18].

An electrochemical immunosensor is commonly designed by sandwiching the analyte of interest between a capture antibody linked to an electrode surface and a signal antibody that generates the analytical response [19,20]. A key consideration is to immobilize the capture antibody in such a manner as to provide an optimal orientation for binding to its analyte, with minimal steric hindrance. The capture antibody may be immobilized via covalent attachment, physical adsorption or electrostatic entrapment in a polymer matrix. This may be achieved using a variety of substances, including Nafion, sol-gels, lipid membranes, conducting polymers and self-assembled monolayers. In previous approaches, a bacterial antibody-binding protein (e.g., protein G) was thiolated so that it could be covalently bonded to a gold electrode (GE). Such proteins will only bind antibodies through the non-antigenic regions, leaving the antigen binding sites of the antibodies available to bind to their target antigens. There have been only a limited number of studies reporting the practical application of protein G [21]. The high affinity of the gold surface facilitates direct formation of a self-assembled layer of thiolated protein G. Following establishment of this layer, the coated GE was incubated in a solution of capture antibody (mAb1) so that these antibodies could attach to the thiolated protein G layer through their non-antigenic regions, leaving their antigen binding sites available for binding of the target analyte with minimal steric hindrance [22,23].

Signal amplification has been used extensively for the development of ultrasensitive amperometric immunoassay methods. In order to meet the increasing demand for early and ultrasensitive detection of tumor markers, three primary signal amplification strategies have been developed, using nanomaterials [24]. The first method involves the use of metal and semiconductor nanoparticles directly as electro-active labels to amplify the electrochemical detection of proteins [25,26]. The second method utilizes nanoparticles as carriers for the loading of a large amount of electroactive species that amplifies the detection signal [27,28]. The third method has been the most extensively employed, and uses enzyme-functionalized nanoparticles as labels. Enhanced sensitivity has been achieved by loading a large amount of enzyme for an individual sandwich immunological reaction event. To date, the use of a nanopolymer to immobilize an enzyme, such as horseradish peroxidase (HRP) as a signal amplifier, had been of particular interest in biosensor design, because of the outstanding optical and electronic performance and biocompatibility.

An Envision antibody complex (EVC) is composed of an IgG signal antibody and several enzymes in a polymer (*i.e.*, connected by a poly-dextran amine skeleton). Since a molecular complex of EVC contains about 100 molecules of HRP and 15 molecules of the secondary antibody [29], the absolute amount of HRP in EVC is much higher than that found with other types of signal antibody. Thus, EVC may be used as a valuable tool for immunohistochemical staining of hard tissues. EVC has recently become available, and has been described as a very sensitive method for development of an immunohistochemical detection system. Since EVC has many HRPs and secondary antibodies, it has significant potential for use in the signal amplification mechanism of an electrochemical immunosensor with a sandwich format. However, there are currently no reports on the actual application of EVC as an enzyme-functionalized label in an electrochemical immunosensor.

EVCs are composed of a long branching polymer associated with vast quantities of HRP enzyme, which can be employed as a linker for the immobilization of AFP signal antibody (AFP Ab2) to produce an EVC-AFP Ab2 signal tag. Our present work on an electrochemical immunosensor for AFP detection, based on nano-gold (AuNPs) and EVC-AFP Ab2 bioconjugates to achieve ultrasensitive signal amplification, was developed to verify the amplification process of enzyme-functionalized nanoparticles. Enhanced detection sensitivity was obtained using multi-labeled bioconjugates, with multiple HRP molecules linked to the EVC-AFP Ab2 signal tag for signal development. Using this approach, we obtained enriched enzyme loading in each sandwich immunological reaction, which subsequently enhanced the electrocatalytic current measured for detection of AFP protein. This simple, cost-effective and sensitive immunosensor would have the ability to detect most cancer biomarker proteins in human serum at levels near or below the normal range, and thus could be used in clinical analysis for a wide range of potential applications.

## 2. Results and Discussion

### 2.1. Morphological Characterization of the Envision Nanocomposite

**Figure 1:**
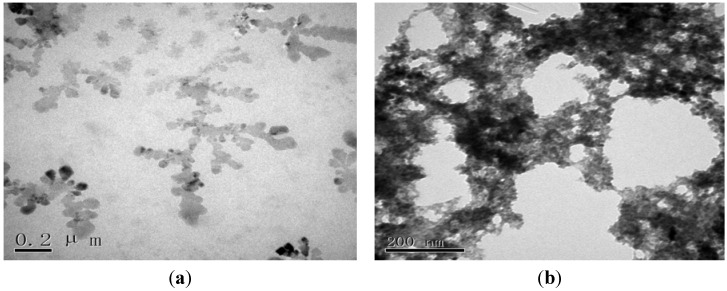
TEM images of (**a**) EVC and (**b**) EVC-AFP Ab2.

**Figure 2:**
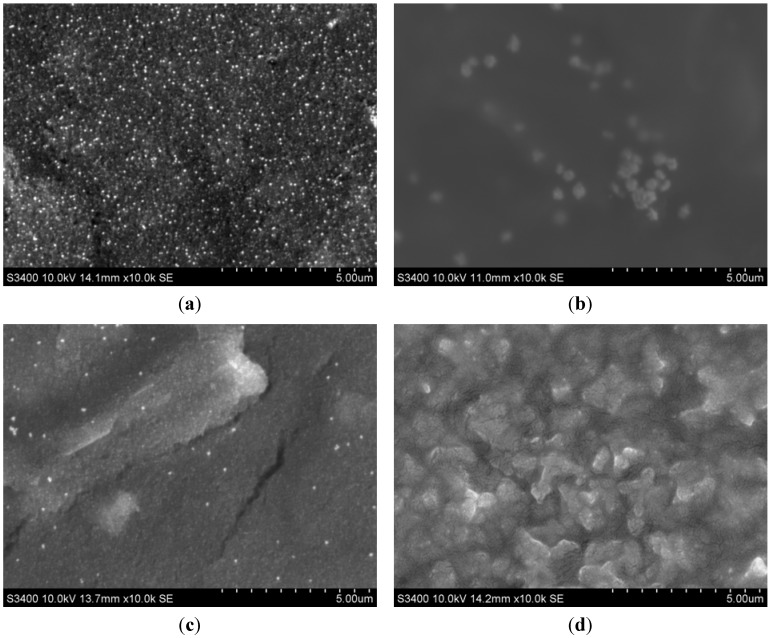
SEM images of (**a**) AuNPs/GE; (**b**) Thiolated protein G/AuNPs/GE; (**c**) AFP Ab1/thiolated protein G/AuNPs/GE; (**d**) Sandwich incubated with EVC-AFP Ab2.

The morphology of the EVC was characterized using transmission electron microscopy (TEM) and scanning electron microscopy (SEM). The TEM image of the EVC (Figure 1) shows that a large amount of HRP (black island shapes) was immobilized on the EVC. After AFP Ab2 was conjugated to the EVC, a “tree-shaped” copolymer was visualized, indicating that AFP Ab2 had likely connected to the skeleton of the EVC (Figure 1b). The SEM image of the AuNPs/GE (Figure 2a) revealed that AuNPs of a homogeneous grain size were uniformly deposited on the surface of the electrode, and were visible as many, small, bright spots. It may be seen in Figure 2b that when the AuNPs were embedded in thiolated protein G, the size of the spots became larger, indicating that the thiolated protein G had become modified by the AuNPs. After reacting AFP Ab1 with thiolated protein G, the corresponding SEM image showed a flat protein membrane spread over the electrode. After the occurrence of the sandwich immune response involving the capture antibody of the sensor, AFP (antigen) and the EVC-AFP Ab2 (signal tag), a porous film was apparent on the surface of the immunosensor (Figure 2d), suggesting that a sandwich immune-complex had been formed.

**Figure 3:**
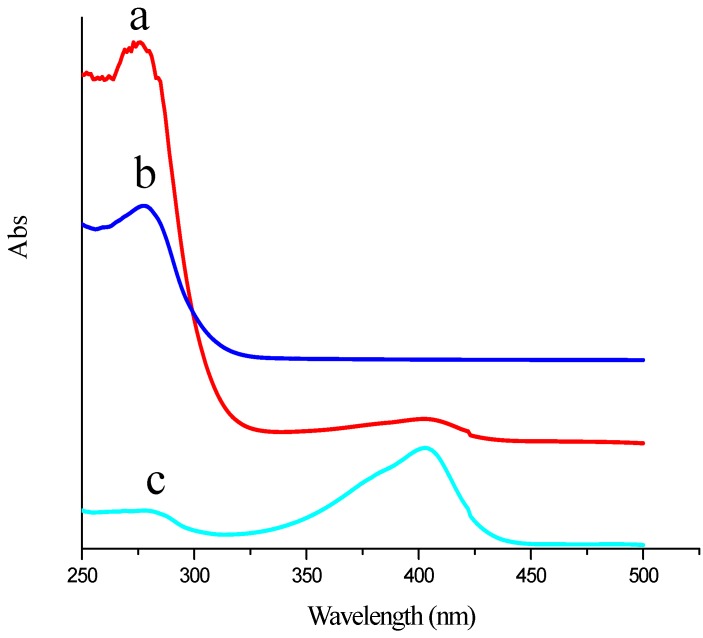
UV-VIS of (**a**) EVC-AFP Ab2; (**b**) AFP Ab2-HRP; (**c**) HRP, respectively.

Ultraviolet-visible (UV-VIS) absorption spectrometry was also employed to characterize the EVC-AFP Ab2. As illustrated by the trace labeled “a” in Figure 3, EVC-AFP Ab2 showed two absorption bands, one at 280 nm that was attributed to AFP Ab2 (see trace “b” obtained for AFP Ab2-HRP), and another at 410 nm that was attributed to HRP (see trace “c” obtained for HRP alone). These results indicate that the EVC had been successfully prepared using AFP Ab2 and HRP. Based on the above results, it may be concluded that EVC-AFP Ab2 had been successfully constructed for use in the detection of AFP.

X-ray fluorescent spectroscopic (XRF) analysis demonstrated that after electrodeposition of AuNPs, characteristic peaks for gold element (Au 4s, Au 4p, Au 4d and Au 4f) appeared between 80 and 90 eV. The intensity of the characteristic peak for sulfur, S_1s_, at 2.4 eV increased after thiolated protein G, AFP Ab1, AFP antigen and EVC-AFP Ab2 were applied to the electrode; this is because all the above proteins contain sulfur. As the immunosensor was modified with the above proteins layer by layer, the sulfur content increased with the concentration of protein.

### 2.2. Electrochemical Characterization of the Immunosensor

**Figure 4:**
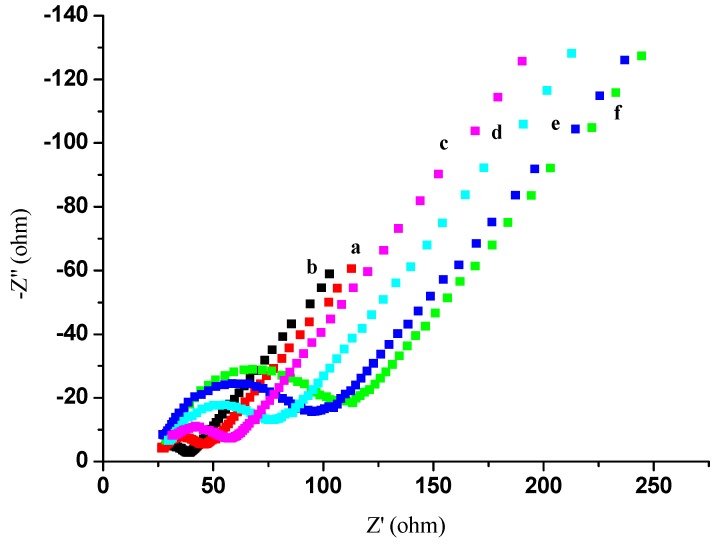
EIS of the different electrodes measured in 5.0 mmol/L [Fe(CN)_6_]^4−/3−^ containing 0.1 mol/L KCl (pH 6.5): (**a**) Bare GE; (**b**) AuNPs/GE; (**c**) Thiolated protein G/AuNPs/GE; (**d**) AFP Ab1/thiolated protein G/AuNPs/GE; (**e**) BSA/AFP Ab1/thiolated protein G/AuNPs/GE; (**f**) AFP antigen/AFP Ab1/thiolated protein G/AuNPs/GE.

Electrochemical impedance spectroscopy (EIS) has been used to study the interfacial properties of modified electrodes [30]. Impedance measurements were performed at a DC potential of 0.23V in 5 mmol/L [Fe(CN)6]^4−/3−^ containing 0.1 mol/L KCl (pH 6.5) in 0.01 mol/L phosphate buffer solution (PBS) as a supporting electrolyte. An alternating voltage (peak-to-peak) of 5 mV was superimposed on the applied DC potential. Nyquist plots were recorded over the frequency range 10 kHz to 0.1 Hz. Simulations were performed using the SIM software program (MEP Instruments Pty Ltd, NSW, Australia) [30]. In EIS, the linear part at low frequencies and the semicircular portion at high frequencies correspond to the diffusion-limited process and the electron transfer-limited process, respectively. The semicircle diameter equals the electron transfer resistance (Ret). Figure 4 shows a series of electrochemical spectra obtained at different stages of the modified electrode in 5 mmol/L [Fe(CN)6]^4−/3−^ containing 0.1 mol/L KCl (pH 6.5). Based on trace “a”, a Ret of approximately 50 $\underset{.}{Ω}$ was estimated. However, after depositing AuNPs on the same electrode (trace “b”), the Ret decreased to 30 Ω because of the ease of electron transfer from [Fe(CN)6]^4−/3−^ by AuNPs. Next, when a thiolated protein G layer was deposited (trace “c”), the Ret increased to approximately 60 Ω, as the thiolated protein G layer constituted a barrier for electron transfer from [Fe(CN)6]^4−/3−^ at the electrode surface. Finally, when 0.5 mg/mL AFP Ab1 was immobilized on the electrode (trace “d”), the Ret increased to approximately 80 Ω. This was ascribed to the non-conducting property of AFP Ab1, hindering the electron transfer reaction. Similarly, the Ret increased to 100 $\underset{.}{Ω}$when bovine serum albumin (BSA) was used to block non-specific binding (trace “e”). After the modified electrode was incubated in 0.2 ng/mL AFP antigen solution, the Ret increased to 115 Ω (trace “f”), as a result of the AFP immunocomplex hindering the electron transfer reaction.

**Figure 5:**
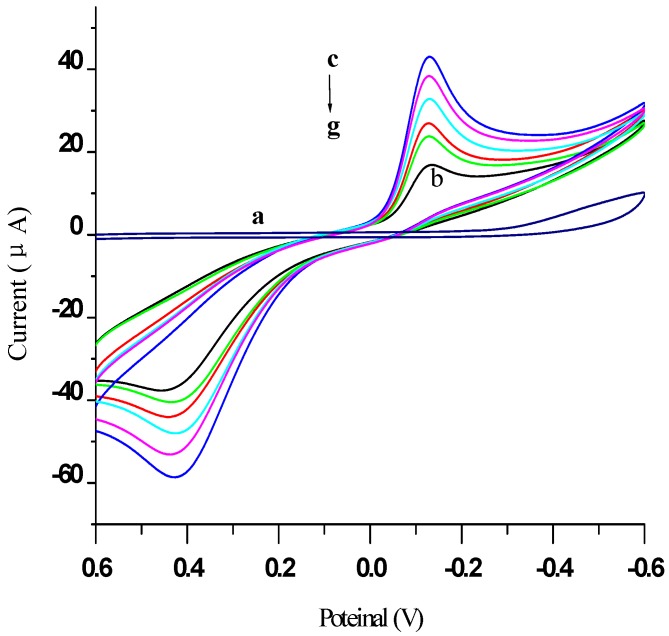
CVs of different modified electrodes measured in: (**a**) 0.01 mol/L PBS containing 0.1 mol/L KCl (pH 7.4); when added 5 mmol/L HQ: (**b**) Bare GE; (**c**) AuNPs/GE; (**d**) Thiolated protein G/AuNPs/GE; (**e**) AFP Ab1/thiolated protein G/AuNPs/GE; (**f**) BSA/AFP Ab1/thiolated protein G/AuNPs/GE; (**g**) AFP antigen/AFP Ab1/thiolated protein G/AuNPs/GE.

Figure 5 illustrates the cyclic voltammograms (CVs) obtained at different stages of the modified electrode, recorded using cyclic voltammetry in PBS (pH 7.4) from −0.6 to 0.6 V (*vs.* the saturated calomel electrode, SCE) at 50 mV/s. As expected, a featureless voltammogram was obtained with a bare GE in PBS (curve “a”). When 5 mmol/L hydroquinone (HQ) was added, a current peak at 0.42 V for the oxidation of HQ to benzoquinone (BQ), and a current peak at −0.12 V for the reduction of BQ to HQ, appeared in trace “b”. Following the deposition of AuNPs on the electrode, the CV obtained (trace “c”) showed a 186% increase in the peak current, as a result of AuNPs facilitating the electron transfer reaction for HQ. After depositing a thiolated protein G layer on the electrode surface, the peak current was found to be 16% smaller (trace “d”). As shown in trace “e”, the peak current was further decreased by 14% after immobilization of AFP Ab1 on the electrode, indicating hindered electron transfer by AFP Ab1. Similarly, a further 15% reduction in the peak current (trace “f”) was observed after BSA was used to block non-specific binding. Finally, after the immunosensor was incubated in an AFP antigen solution (trace “g”), the peak current declined by 18% compared to that in trace “f”, as the immunocomplex hindered tunneling mass and electron transfer at the electrode. These results are thus in agreement with those of EIS.

### 2.3. Optimization of Experimental Conditions

**Figure 6:**
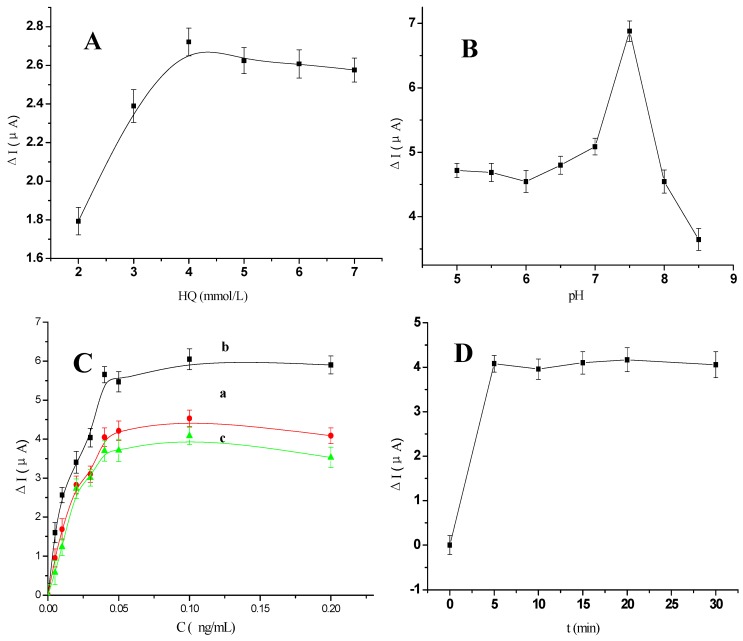
(**A**) The effect of the different concentration of HQ from 2 mmol/L to 7 mmol/L; (**B**) The pH dependency of the anodic peak currents responses for the immunosensor; (**C**) The effect of the different volume ratio of EVC and AFP Ab2 (**a**) 0.1:1, (**b**) 1:1 and (**c**) 100:1 ([AFP Ab2] = 50 μg/mL); (**D**) The effect of the different incubation time of EVC-AFP Ab2 after the immunosensor incubated with 0.2 ng/mL AFP solution. All experiments scan rate 50 mV/s, in 0.01 mol/L PBS/0.1 mol/L KCl (pH 7.4) added 5 mmol/L HQ.

As illustrated in Figure 6A, the concentration of HQ was optimized by varying its concentration from 2 mmol/L to 7 mmol/L. The current value increased to a maximum when HQ reached a concentration of 5 mmol/L. Therefore, 5 mmol/L was chosen as the optimum concentration of HQ for use in further experiments.

The effect of buffer pH on the behavior of the immunosensor was investigated over the pH range 5.0 to 8.5, using 0.2 ng/mL AFP. In these experiments, the HQ acted as an electron transfer mediator that could produce background current (I_0_); when the samples were added, its oxidation current changed accordingly. We used the difference in the oxidation current (ΔI) for quantification: the oxidation current of HQ was initially measured before samples were added (I_0_), and then re-measured after additions were made (I_i_). The difference (ΔI) between I_i_ and I_0_ was then plotted against each parameter being studied. As shown in Figure 6B, as the pH was increased from 5.0 to 8.0, the current initially increased to reach a maximum value at pH 7.5, and then decreased as pH was further elevated to pH 8.5. The stability of an antibody-antigen complex is known to be pH dependent, and strong acidic medium could destabilize this [31,32]. In view of the fact that a highly acidic or alkaline solution would likely damage the bioactivity of immobilized proteins, pH 7.5 was chosen as the optimal pH for detection, in order to obtain a high sensitivity.

When using the EVC-AFP Ab2 as a detection antibody, the ratio of EVC to AFP Ab2 chosen will have an effect on the interaction efficiency between the detection antibody and the AFP antigen. To determine the optimal ratio of EVC to AFP Ab2, various volume ratios were used for the preparation of EVC-AFP Ab2: 0.1:1, 1:1 and 100:1. As shown in Figure 6C, ΔI increased to a maximum when the ratio of EVC to AFP Ab2 reached 1:1. Thus, an equal ratio was chosen as the optimal volume ratio to prepare EVC-AFP Ab2.

The incubation time for the immunoassay is another important factor influencing the reaction between an antigen and an antibody. Figure 6D shows the dependence of ΔI on incubation time. The modified electrode was immersed in EVC-AFP Ab2 (EVC:AFP Ab2 = 1:1, by volume) for 0, 5, 15, 20, 30 and 35 min. The current response was found to be increased at an incubation time of 5 min, but then tended to level off. Thus, 5 min was selected as the optimal incubation time for subsequent studies.

### 2.4. Characterization of Signal Amplification by EVC-AFP Ab2 Using Different Labels

**Figure 7:**
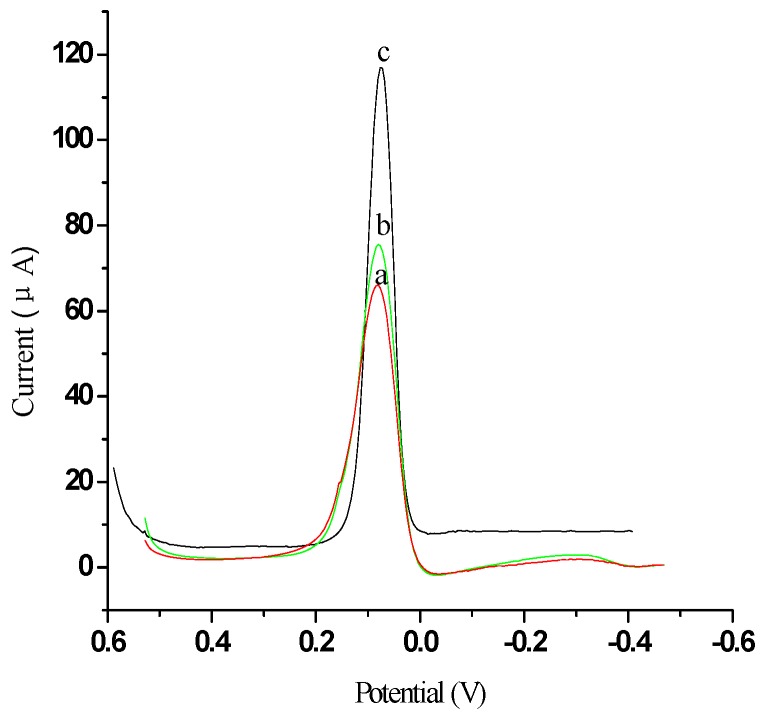
The characterization of signal amplification by different labels: (**a**) Bare GE; (**b**) Modified AFP Ab2-HRP; (**c**) EVC-AFP Ab2. Scan rate 50 mV/s, in 0.01 mol/L PBS/0.1 mol/L KCl (pH 7.4) and added 5 mmol/L HQ.

To investigate the advantages of electrochemical ELISA using EVC bioconjugated detection antibodies, we constructed two types of detection antibody as labels, namely AFP Ab2-HRP and EVC-AFP Ab2. In this experiment, 5 pg/mL AFP was used for evaluation of the electrochemical signal response. Judgment of the response was based on the current change (ΔI) between the reduction peak current for the oxidation product of HQ, generated in the electrochemical immunosensor, and the background current of the GE (Figure 7a). As shown in Figure 7, the use of EVC-AFP Ab2 as the detection antibody (ΔI = 43.82 µA, Figure 7c) resulted in a much higher current response (approximately 4.5-fold greater) than that obtained using AFP Ab2-HRP (ΔI = 9.78 µA, Figure 7b). This indicates that an immunosensor based on EVC-AFP Ab2 can generate substantial signal amplification, allowing the development of ultrasensitive amperometric immunoassays. A possible reason for this advantage may be that the amount of HRP bound was much higher for EVC-AFP Ab2 than for AFP Ab2-HRP. When one EVC-AFP Ab2 detection antibody reacted with the antigen, numerous HRPs participated in the HRP enzymatic reaction, facilitating the oxidation of HQ by H_2_O_2_ and producing more signal species for improved sensitivity.

### 2.5. Calibration Curve of the Immunosensor

**Figure 8:**
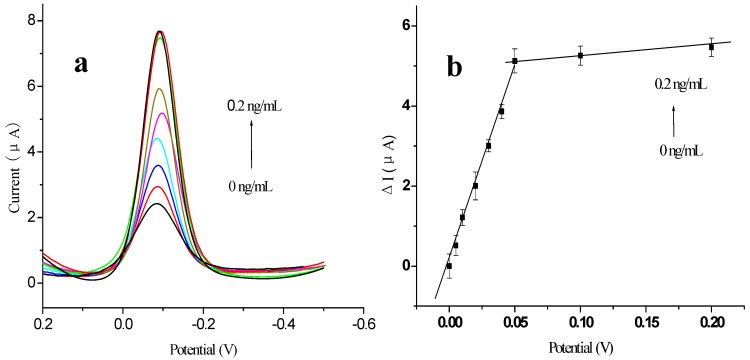
Voltammogram of modified electrode using different concentrations of antigen (0 ng/mL, 0.005 ng/mL, 0.01 ng/mL, 0.02 ng/mL, 0.03 ng/mL, 0.04 ng/mL 0.05 ng/mL, 0.1 ng/mL and 0.2 ng/mL, respectively): (**a**) DPV curves; (**b**) plot of ΔI *vs.* the concentrations of AFP. All experiments were carried out in 0.01 mol/L PBS/0.1 mol/L KCl (pH 7.4) and added 5 mmol/L HQ, at a potential range of 0.2 V to −0.5 V, *vs.* SCE. Scan rate: 50 mV/s.

The calibration curve for the detection of AFP was obtained using the prepared immunosensor under the optimal experimental conditions (Figure 8). When antigens bound with the antibodies immobilized on the electrode, the antigen–antibody complexes coating the surface of the electrode inhibited electron transfer. The change in the reduction peak current response (ΔI) of the immunosensor was found to be linearly proportional to the AFP concentration in the range 0.005 to 0.2 ng/mL, with two distinct linear ranges evident. The detection limit of the method was 2 pg/mL AFP (signal-to-noise ratio, S/N = 3). The correlation coefficient was 0.998 for both linear ranges. It is likely that the calibration curve presented with two slopes because the amount of antibody immobilized on the electrode surface was constant. As the antigen concentration increased, the active sites of the antibodies immobilized on the electrode surface became fewer, leading to a decrease in the sensitivity [33]. Table 1 provides a comparison of the analytical properties of various AFP immunosensors and immunoassays.

**Table 1:** Comparison of analytical properties of various AFP immunosensors or immunoassays.

Assay method	Linear range (ng·mL^−1^)	LOD (ng·mL^−1^)	Signal antibody	Reference
ELISA	0.7–15	0.2	HRP labeled AFP Ab2	The used Kit’s instructions
Electrochemical immunoassay	0.1–30.0	0.018	label-free	[35]
	0.02–3.5	0.096	DNA modified gold nanoparticles	[36]
Chemiluminescence	0.01–100	0.005	SiO_2_/HRP–antibody	[37]
Electrochemiluminescence immunoassay	0.05–50	0.03	Ru-silica@Au–antibody	[7]
Electrochemical ELISA	0.005–0.2	0.002	EVC-AFP Ab2	This work

### 2.6. Regeneration of the Immunosensor

Regeneration is an important factor required for the practical application of an immunosensor. To further evaluate the possibility that the developed electrochemical immunosensor could be used for testing real-life human serum samples, its performance was compared with that of an ELISA reference method, for the analysis of six clinical serum specimens supplied by the Nanfang Medical Hospital, China. The results are listed in Table 2. The relative deviation of the measured data was between 2.3% and 4.5%, indicating good agreement between the two analytical methods.

**Table 2:** Experimental results comparison of two methods obtained in serum samples (n = 3).

Serum samples	1	2	3	4	5	6
ELISA (ng/mL)	ND	10	20	40	ND	ND
Immunosensor (ng/mL)	2.1	10.5	21	42	0.2	0.1
Relative deviation (%)	2.3	4.5	2.4	3.1	3.2	3.2

Note: *ND: not detected

## 3. Experimental Section

### 3.1. Apparatus and Regents

AFP’s ELISA kit was supplied by Huaan Magnech Bio-tech Co., Ltd (Beijing, China). Envision^TM^ Detection Kit was supplied by Gene Tech Company Limited (Shanghai, China). Bovine serum albumin (BSA, 96%–99%), and hydrogen tetrachloroaurate(Ⅲ) tetrahydrate (HAuCl_4_), HQ and H_2_O_2_ were obtained from Sinopharm Group Chemical Reagent Company Ltd (Shanghai, China). Phosphate buffer saline (PBS, 0.01 mol/L pH 7.4) containing 0.1 mol/L KCl was used to prepare the protein solution. The blocking buffer solution was PBS (pH 7.4) containing 3% (w/v) BSA. Tween-PBS buffer (0.01 mol/L pH 7.4) containing 0.05% (w/v) Tween-20 was used as the washing solution. All other reagents were of analytical grade and were used without further purification. Double distilled water was used throughout the study. All experiments were carried out at room temperature.

Differential pulse voltammetry (DPV) was performed using an electrochemical analyzer CHI 660 electrochemical analyzer (Shanghai Chen hua Instrumental Corp, Shanghai, China). A conventional three-compartment electrochemical cell consisting of a platinum wire auxiliary electrode, a saturated calomel reference electrode and a modified GE (2 mm diameter) as a working electrode was used. The sizes of the nanoparticles were estimated by TEM (H-7650, Hitachi Instruments, Tokyo, Japan). The surface topographic features and composition of different modified electrodes were characterised using SEM (S3400N, Hitachi Instruments, Tokyo, Japan), UV-VIS and XRF.

### 3.2. Preparation of Envision-AFP Antibody Copolymer (EVC-AFP Ab2)

Equal volumes of AFP Ab2 and Envision antibody complex were mixed and stored overnight at 4 °C. After centrifuging, the EVC-AFP Ab2 was stored at 4 °C until further use.

### 3.3. Preparation of Thiolated Protein G Modified Au Electrode

Thiolated protein G solution was prepared as described by Fowler *et al.* [23]. In the direct formation of a protein G layer on a gold electrode surface, the amine groups associated with a series of lysine residues in the protein G molecule were first converted to thiol groups using 2-iminothiolane. A 10-fold molar excess of 2-iminothiolane prepared in degassed PBS was reacted with protein G that had been dissolved in degassed PBS for 30 min at 4 °C. Excess 2-iminothiolane was immediately removed by centrifugal filtration, and the protein was concentrated to 100 μg*/*L.

### 3.4. Fabrication of the Immunosensor

As previously reported [38], before modification, GE was polished with 0.05 μm alumina powder, rinsed successively in an ultrasonic bath with double distilled water, absolute alcohol and double distilled water for 5 min, respectively. Then GE was soaked in the solution (H_2_SO_4_:H_2_O_2_ = 7:3, by volume) for 15 min. The electrode was electrochemically treated by cycling the potential between −0.3 and +1.5 V in the 0.1 mol/L H_2_SO_4_. Finally, the electrode was rinsed with double distilled water, dried in a nitrogen stream and was ready for use.

**Figure 9:**
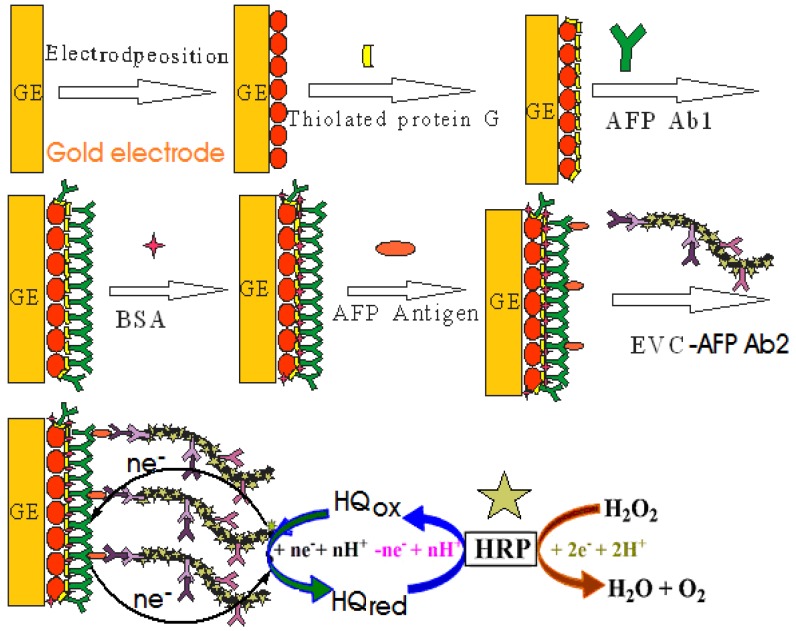
Schematic illustration of the fabrication and detection procedure of the immunosensor.

The polished gold electrode was immersed in the as-prepared 1 mmol/L HAuCl_4_ solution, and the electrodeposition was performed by applying a constant voltage of −0.5 V for 20 s. After electrodeposition, the modified AuNPs/GE was removed from the solution and rinsed with double distilled water, then dried in air at room temperature. Subsequently, the modified electrode immersed in thiolated protein G solution at 4 °C overnight, the modified electrode (thiolated protein G/AuNPs/GE) was removed from the solution and rinsed with double distilled water. Next, 20 μL of anti-AFP Ab1 solution was applied to the modified electrode surface and kept at 4 °C for 2 h to produce an AFP Ab1/thiolated protein G/AuNPs/GE. Finally, this modified electrode was incubated in 3% BSA for 30 min in order to block possible remaining active sites and avoid non-specific adsorption. The finished immunosensor was stored at 4 °C when not in use. The fabricated procedure and detection principle of the immunosensor is summarized in Figure 9.

### 3.5. Electrochemical Measurements of AFP

The prepared immunosensor was incubated in 20 μL incubation solution containing different concentration of AFP at 37 °C for 30 min. After the residual was removed with PBS, the modified electrode was incubated in 20 μL different concentrations of EVC-AFP Ab2 at 37 °C for 5 min. After the residual was removed with Tween-PBS, DPV from 0.2 to −0.6 V (*vs.* SCE) was recorded in 0.1 mol/L PBS (pH 7.4). The measurement principle was based on the inhibition of the response current of hydroquinone in the PBS after the formation of antigen–antibody complex, which was directly proportional to the concentration of AFP.

## 4. Conclusions

In this work, a novel signal amplification strategy for construction of an electrochemical sandwich immunosensor was developed based on EVC-AFP Ab2. The results described above demonstrate that amplification proceeds via a large amount of HRP binding to EVC-AFP Ab2 signal tag. The electrochemical reaction between HQ and H_2_O_2_ catalyzed by HRP can produce obvious catalyzed current on the surface of the immune electrode, which significantly extends sensitivity, leading to an unprecedented detection limit for AFP of 5 pg/mL. Multi-functionality of an EVC-AFP Ab2 was demonstrated with the application to the immunosensor. Meanwhile, in the fabrication of the immunosensor electrode, AuNPs-thiolated protein G nanocomposite was employed to immobilize antibody, which can offer a large specific surface area and biocompatible microenvironment for the immobilization of a larger amount of antibodies. Furthermore, a thiolated protein G layer, through its nonantigenic regions, leaves antigen binding sites available with minimal steric hindrance for binding of target analyte. Therefore, the proposed immunosensor presented some advantages, including a simple and controllable fabrication process, high sensitivity, low detection limit, satisfactory reproducibility and cost-effectiveness. The proposed immunosensor could provide a new approach to detection in clinical diagnosis.
